# Traditional Chinese medicine Danggui Buxue Tang inhibits colorectal cancer growth through induction of autophagic cell death

**DOI:** 10.18632/oncotarget.19902

**Published:** 2017-08-03

**Authors:** Shun-Ting Chen, Tzung-Yan Lee, Tung-Hu Tsai, Yu-Chuen Huang, Yin-Cheng Lin, Chin-Ping Lin, Hui-Ru Shieh, Ming-Ling Hsu, Chih-Wen Chi, Ming-Cheng Lee, Hen-Hong Chang, Yu-Jen Chen

**Affiliations:** ^1^ Department of Chinese Medicine, Taipei Buddhist Tzu Chi Hospital, Buddhist Tzu Chi Medical Foundation, New Taipei City 23142, Taiwan; ^2^ Graduate Institute of Traditional Chinese Medicine, School of Chinese Medicine, College of Medicine, Chang Gung University, Taoyuan 33302, Taiwan; ^3^ Graduate Institute of Clinical Medical Science, College of Medicine, Chang Gung University, Taoyuan 33302, Taiwan; ^4^ Institute of Traditional Medicine, School of Medicine, National Yang-Ming University, Taipei 11221, Taiwan; ^5^ School of Chinese Medicine, China Medical University, Taichung 40402, Taiwan; ^6^ Department of Medical Research, China Medical University Hospital, Taichung 40402, Taiwan; ^7^ Department of Medical Research, Mackay Memorial Hospital, New Taipei City 25160, Taiwan; ^8^ Department of Research, Taipei Buddhist Tzu Chi Hospital, Buddhist Tzu Chi Medical Foundation, New Taipei City 23141, Taiwan; ^9^ School of Post-Baccalaureate Chinese Medicine, College of Chinese Medicine, and Research Center for Chinese Medicine and Acupuncture, China Medical University, Taichung 40402, Taiwan; ^10^ Department of Chinese Medicine, China Medical University Hospital, Taichung 40402, Taiwan; ^11^ Department of Radiation Oncology, Mackay Memorial Hospital, Taipei 10449, Taiwan; ^12^ Depatment of Chemical Engineering, National United University, Miaoli 36003, Taiwan

**Keywords:** Danggui Buxue Tang, colorectal cancer, autophagy, traditional Chinese medicine, mammalian target of rapamycin (mTOR)

## Abstract

**Purpose:**

The induction of autophagic cell death is an important process in the development of anticancer therapeutics. We aimed to evaluate the activity of the ancient Chinese decoction Danggui Buxue Tang (DBT) against colorectal cancer (CRC) and the associated autophagy-related mechanism.

**Materials and methods:**

CT26 CRC cells were implanted into syngeneic BALB/c mice for the tumor growth assay. DBT extracts and DBT-PD (polysaccharide-depleted) fractions were orally administered. The toxicity profiles of the extracts were analyzed using measurements of body weight, hemogram, and biochemical parameters. The morphology of tissue sections was observed using light and transmission electron microscopy. Western blotting and small interference RNA assays were used to determine the mechanism.

**Results:**

DBT-PD and DBT, which contained an equal amount of DBT-PD, inhibited CT26 syngeneic tumor growth. In the tumor specimen, the expression of microtubule-associated proteins 1A/1B light chain 3B (LC3B) was upregulated by DBT-PD and DBT. The development of autophagosomes was observed via transmission electron microscopy in tumors treated with DBT-PD and DBT. *In vitro* experiments for mechanism clarification demonstrated that DBT-PD could induce autophagic death in CT26 cells accompanied by LC3B lipidation, downregulation of phospho-p70^s6k^, and upregulation of Atg7. RNA interference of Atg7, but not Atg5, partially reversed the effect of DBT-PD on LC3B lipidation and expression of phospho-p70^s6k^ and Atg7. The changes in ultrastructural morphology and LC3B expression induced by DBT-PD were also partially blocked by the knockdown of Atg7 mRNA.

**Conclusion:**

DBT induced autophagic death of colorectal cancer cells through the upregulation of Atg7 and modulation of the mTOR/p70^s6k^ signaling pathway.

## INTRODUCTION

Colorectal cancer (CRC) is a common and lethal disease; furthermore, the worldwide incidence and mortality rates are increasing rapidly [1]. Age is a major risk factor for sporadic CRC, the incidence of which is uncommon before the age of 40, begins to increase significantly between the ages of 40 and 50, and shows an increase in age-dependent incidence rates with each succeeding decade [2]. Since the mid-1980s, death rates from CRC have declined progressively in the United States and the rest of the world [3, 4]. However, the overall survival rates of CRC are still unsatisfactory, especially for the late-stage disease [5].

Despite recent advances in cytotoxic and targeted therapy, the development of resistance to chemotherapy remains one of the greatest challenges in the long-term management of incurable metastatic diseases which eventually contributes to death [6]. To overcome resistance and advance the fight against this disease, the achievement of better treatment responses and longer patient survival is essential. New therapeutic interventions such as BRAF inhibitors, MEK inhibitors, and anti-programmed death 1 immune checkpoint inhibitors have been actively studied for the treatment of CRC [7]. The PI3K/AKT/mTOR pathway, which is an important intracellular signaling pathway in cell cycle regulation, is directly related to cellular quiescence, proliferation, cancer, and longevity. Proteins that regulate the mammalian target of rapamycin (mTOR), as well as certain targets of the mTOR kinase, are overexpressed or mutated in cancer. Rapamycin analogs, have been shown to inhibit the growth of cell lines derived from multiple tumor types *in vitro* and tumor models *in vivo* [8]; clinical trials have also indicated that rapalogs may be useful for the treatment of subsets of certain types of cancer [9].

Danggui Buxue Tang (DBT), an herbal decoction, has been used in Chinese medicine to enhance qi and blood circulation in Asian patients [10] for more than 2000 years. It consists of *Astragali* Radix (AR) and *Angelicae sinensis* Radix (ASR) in the ratio of 5:1. Previous studies have found that DBT could modulate hematopoietic function [11–13], osteoproliferation, and differentiation [14, 15]. Estrogenic effects were not only demonstrated in the MCF-7 breast cancer cell line [16], but also in human trials, which revealed that DBT enhanced the quality of life for postmenopausal women by decreasing hot flashes and night sweats [17]. Other pharmacological activities, such as an antifibrotic effect in rat lung [18], a decrease in angiogenesis [19], and a reduction of oxidative stress in rat liver fibrosis [20], have also been reported. We have previously reported that DBT was a chemoradiotherapy sensitizer with antineoplastic effects in CT26 cancer cells, particularly the polysaccharide-depleted fraction (DBT-PD) [21]. Therefore, DBT may be considered a potential new therapeutic option with a novel mechanism for the treatment of CRC. However, the pathways through which this action occurs require further investigation.

Autophagy (from the Greek for “the eating of oneself”) refers to the evolutionarily conserved process, which regulates the turnover of cellular constituents and occurs during development or as a response to stress. Autophagy is a dynamic, multi-step process that can be modulated at several steps, both positively and negatively. Autophagic flux refers to the complete process of autophagy including the delivery of cargo to lysosomes and its subsequent breakdown and recycling. Autophagic cell death, or more properly autophagy-associated cell death, represent the phenotypic defects arise due to the modulation of autophagy [22]. Macroautophagy is the major catabolic pathway of energy generation and is responsible for the rescue of damaged organelles during periods of stress or nutrient deprivation. During the process, the bulk double-membrane vesicles (autophagosome) engulf larger cytoplasmic proteins and organelles such as mitochondria, fractures ER, and peroxisomes. Then autophagosome fuses with lysosomes to form as a single layer autolysosome for further degradation which could be blocked by chloroquine (CQ, acidification inhibitor) or E64D (inhibitor of lysosomal proteases) [22]. The formation of the autophagosome is ATP-dependent and induced by stress conditions, such starvation or hypoxia. The lack of amino acids and growth factors regulates the mTOR/p70^s6k^ signaling pathway to induce the process of autophagy [23]. The BH3 domains of BNIP3 and BNIP3L expressed in hypoxia displace Beclin1 from Bcl-2 or Bcl-xL, which leads to autophagy [24]. Nucleation is the first step of autophagy in which the isolation membrane is formed and class III phosphatidylinositol-3-kinase (PI3K) is the main protein complex required for this process. Elongation of phagophore is related to the ATG12 conjugation system, which involves the proteins ATG12, ATG5, and ATG16L that create a multimeric complex [25]. The LC3 complex (the mammalian homolog of yeast ATG8) is necessary to close the autophagosome and a key complex in the final step of autophagosome formation. The cytoplasmic LC3 is post-translationally modified to LC3-I and then LC3-II in a series of steps that begin with cleavage by Atg4, followed by ubiquitination reactions that transiently link it with Atg7 and Atg3, and finally lipidation [26]. Adaptor protein p62 directly binds to ubiquitinated proteins and acts as a receptor for ubiquitinated proteins. By binding to LC3-II, p62 facilitates autophagy by localizing in autophagic compartments, transporting ubiquitinated proteins and organelles for degradation [27].

In this study, we examined the effect of DBT and DBT-PD on the growth of CRC cells and clarified the mechanism involved in cell death.

## RESULTS

### Antitumor activity of DBT and DBT-PD

**Figure 1:**
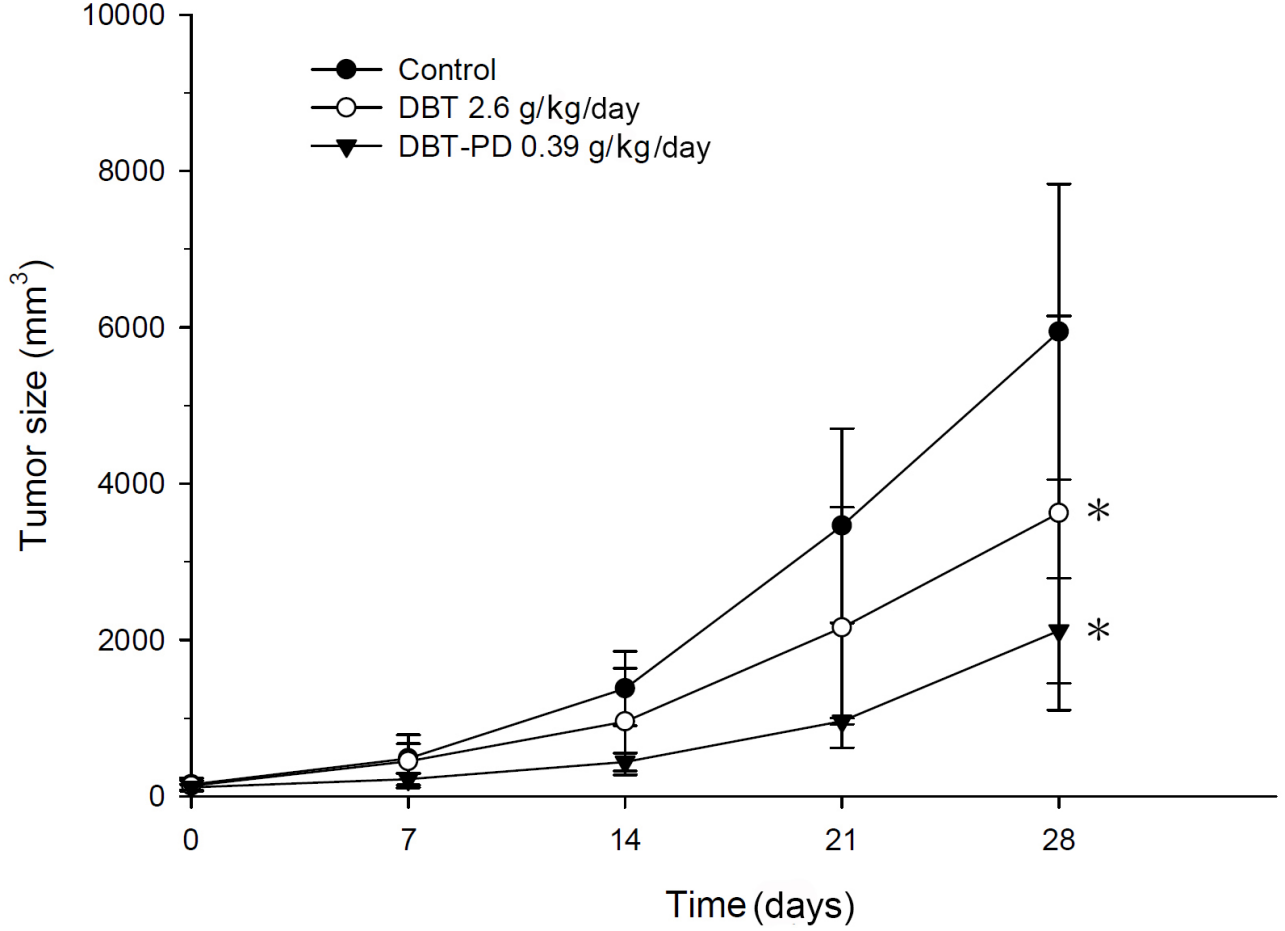
Tumor size in BALB/c mice implanted with CT26 colorectal adenocarcinoma cells Both DBT 2.6 g/kg/day (○) and DBT-PD 0.39 g/kg/day (▼) produced better inhibition of tumor growth than that observed in the control group (●) (*p*<0.05 *). DBT-PD demonstrated the most dramatic efficacy of these three groups, although the components of polysaccharide-depleted fraction in 2.6 g DBT were equal to that in 0.39 g DBT-PD.

In our previous study, we determined that the amount of active ingredient in 2.6 g DBT decoction was equal to 0.3 g DBT-PD [21]. We evaluated the antitumor effect of both DBT and DBT-PD, and found that both DBT and DBT-PD inhibited the growth of colorectal adenocarcinoma *in vivo* (Figure 1). The inhibitory effect of DBT-PD was greater than that of DBT (*p*<0.05).

### Changes in body weight and liver and renal function

**Figure 2:**
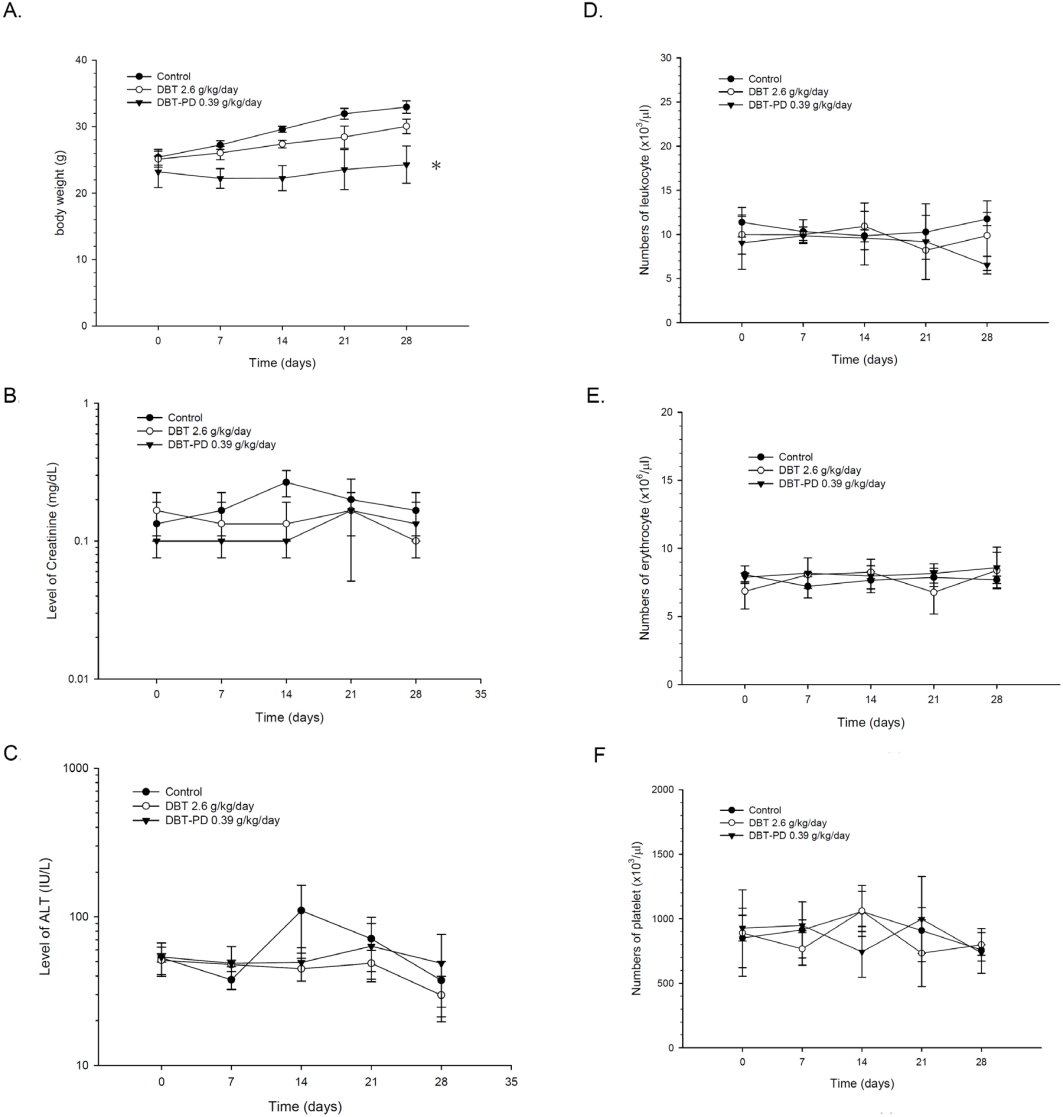
Changes in body weight, serum chemistry, and blood cells of BALB/c mice implanted with CT26 colorectal adenocarcinoma cells Although a mild decrease of body weight in the DBT-PD group was observed (*p*<0.05 *), no significant differences in plasma ALT, creatinine levels, and blood cell counts were observed (*p*>0.05).

No significant differences were observed in the body weight between the control and DBT-treated groups, but a mild decrease occurred in the DBT-PD group (Figure 2A) (*p*<0.05). There were no significant differences in the plasma creatinine or ALT levels between the control, DBT or DBT-PD groups. (Figure 2B and 2C) (*p*>0.05).

### Blood cell count profiles

The number of leukocytes (Figure 2D), erythrocytes (Figure 2E), and platelets (Figure 2F) did not change after treatment of DBT or DBT-PD during the 4-week experimental period (*p*>0.05).

### Morphological examination in tumor specimens

**Figure 3:**
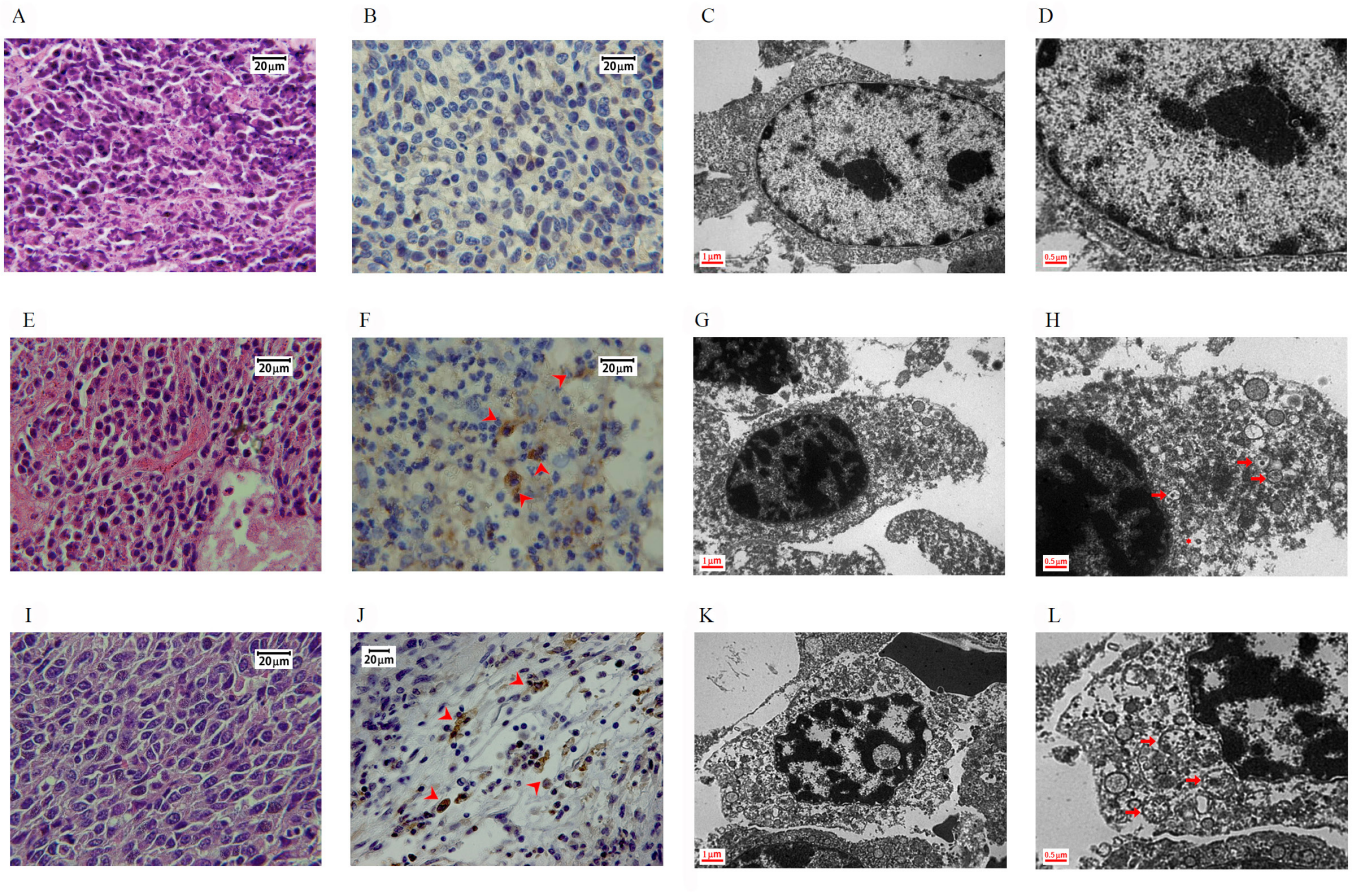
Morphology of BALB/c mice implanted with CT26 colorectal adenocarcinoma cells Images **(A** to **D)**: control group; **(E** to **H)**: 2.6 g/kg DBT-treated group; **(I** to **L)**: 0.39 g/kg DBT-PD-treated group. Hematoxylin and eosin staining showed a more condensed nucleus in the control group (Figure 3A) than in the treatment groups (Figure 3E and 3I). After the immunohistochemistry staining for LC3 protein, both treatment groups (Figure 3F and 3J) showed more brown color (arrowhead) than the control group (Figure 3B), which was and indicator of the activity of the LC3 protein. When examined under a transmission electron microscope (TEM), both the DBT- and DBT-PD-treatment groups showed the presence of autophagosomes (arrow), including the appearance of multiple cytoplasmic vacuoles with double-layered membranes (Figure 3G, 3H, 3K, and 3L), which were autophagosomes that contained degraded organelles, such as markedly swollen mitochondria. Some fusion vacuoles were autolysosomes (asterisk).

Hematoxylin and eosin staining demonstrated more condensed nuclei and irregular shapes in the control group than in the treated groups (Figure 3A, 3E, and 3I). Immunohistochemical (IHC) staining revealed that LC3 protein expression increased in the DBT and DBT-PD-treated groups (Figure 3F and 3J), with no obvious expression in control (Figure 3B). The percentage of LC3B positive cells under IHC stain (Table 1) between control, DBT or DBT-PD-treated groups were statistically significant difference (*p<0.01*). The LC3B expression levels were increased from 0.51% in control group to 46.46 and 49.84% in DBT and DBT-PD-treated groups, respectively. To confirm hyperactive autophagy after the drug treatment, we detected autophagic vesicles via transmission electron microscopy (TEM). Both the DBT- and DBT-PD-treated groups showed the presence of autophagosomes, including the appearance of multiple cytoplasmic vacuoles with double-layered membranes (Figure 3G, 3H, 3K and 3L). Vacuoles that resembled autophagosomes and contained the remnants of degraded organelles were observed. A marked swelling of the mitochondria with vacuolization also observed. Some attached vacuoles were similar to the fusion of an autophagosome and lysosome.

**Table 1:** Percentage of LC3B positive cells using IHC stain in CT26 adenocarcinoma tumor specimens

Control group	2.6 g/kg DBT treated group	0.39 g/kg DBT-PD treated group
0.51 ± 0.20%	46.46 ± 4.99% **	49.84 ± 8.08%**

Significant difference between control and DBT or DBT-PD treated group are indicated as ** *p<0.01*.

### Autophagy-related protein detection and signaling pathway elucidation

**Figure 4:**
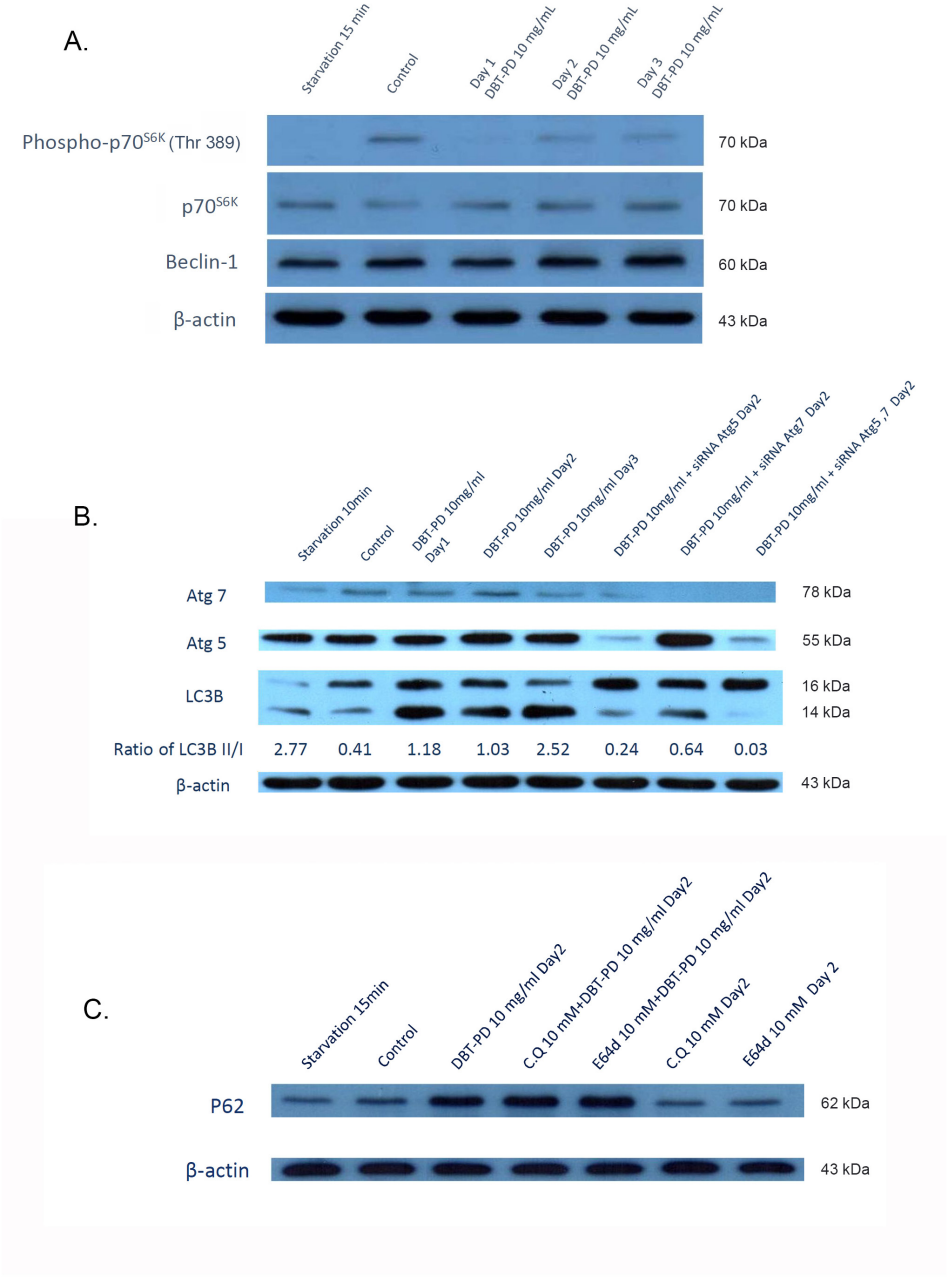
Western blot of CT26 colorectal adenocarcinoma cells after treatment of 10 mg/mL DBT-PD In **(A)**, after the treatment with DBT-PD, phospho-p70^S6K^ decreased dramatically and LC3-I was increase of amount with sequentially transformed to LC3-II. Atg5, Atg7 **(B)** and p62 **(C)** were activated after treated of DBT-PD but not in chloroquine (CQ) and E64D groups to p62 proteins.

In our previous publication, the treatment of 10 mg/mL DBT-PD inhibited the growth of CT26 adenocarcinoma cells and sensitized the cells to chemoradiotherapeutic effects [21]. The mechanism of DBT-PD-induced CT26 cell death was found to be autophagy-associated. Therefore, 10 mg/mL DBT-PD was used to investigate the possible signaling mechanisms associated with autophagy *in vitro*. Autophagy-related proteins such as p70^s6k^, phospho-p70^s6k^, Beclin-1, Atg5, Atg7, LC3-I, LC3-II and p62 were detected by western blot (Figure 4A, 4B and 4C). Treatment with DBT-PD markedly decreased phospho-p70^s6k^ (Figure 4A) and activated Atg5 and Atg7 (Figure 4B). These changes also occurred under serum deprivation conditions. LC3-I was sequentially converted to LC3-II in the presence of DBT-PD and increase the mounts of LC3-I and LC3-II dramatically, the ratio of LC3-II/I elevated from 0.41 to 2.52 on day 3 (Figure 4B). Compared to the control group, p62 proteins also activated after treated by DBT-PD (Figure 4C). This indicated that DBT-PD induced the autophagic flux might be through the mTOR/p70^s6k^ signaling pathway in CT26 cells.

**Figure 5:**
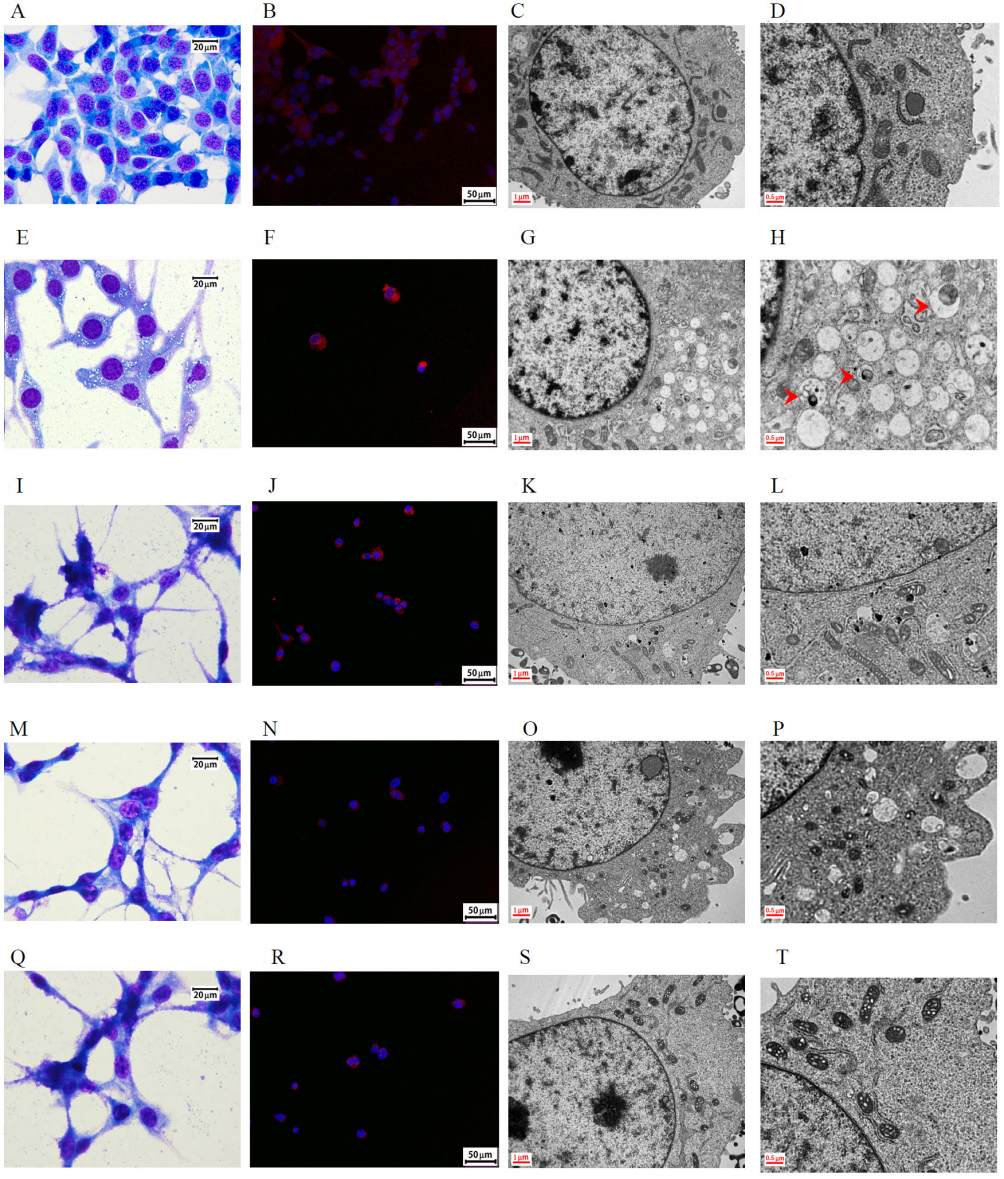
Morphology of CT26 colorectal adenocarcinoma cells after treatment with 10 mg/mL DBT-PD for 48 h Images **(A** to **D)**: control group; **(E** to **H)**: 10 mg/mL DBT-PD-treatment, **(I** to **L)**: Atg5 siRNA plus DBT-PD treatment; **(M** to **P)**: Atg7 siRNA plus DBT-PD treatment; **(Q** to **T)**: Atg5 and Atg7 siRNA plus DBT-PD treatment. Following Liu's staining, abundant cytoplasmic vacuoles were observed in CT26 cells treated with 10 mg/mL DBP-PD relative to control cells (Figure 5A and 5E), but not in cells transfected with siRNA to Atg5 and Atg7 (Figure 5I, 5M and 5Q). The LC3 protein was expressed diffusely in untreated cells and transformed to a punctate distribution pattern of intense acridine orange in DBT-PD-treated cells; however, this phenomenon disappeared after the transfection of Atg5 and Atg7 siRNA (Figure 5B, 5F, 5J, 5N and 5R). When compared to the treatment group alone, the siRNA-treated groups filed to form a complete autophagosome (arrowhead), although the phagophore (arrow) formation had already commenced (Figure 5D, 5H, 5L, 5P and 5T).

Similar results with respect to the morphological changes were observed using Liu’s stain, immunofluorescence staining, and TEM. Light microscopy analysis of CT26 cells treated with DBT-PD showed an abundance cytoplasmic vacuoles compared with the control cells (Figure 5A and 5E), but not in cells treated with Atg5, Atg7, or Atg5 and Atg7 siRNA (Figure 5I, 5M and 5Q). The protein LC3 was diffusely expressed in untreated cells (Figure 5B), which changed to a punctate distribution of intense red fluorescence in DBT-PD treated cells (Figure 5F), but this phenomenon disappeared after the treatment with Atg5, Atg7, or Atg5 and Atg7 siRNA (Figure 5J, 5N and 5R). When compared with the treatment group (Figure 5G and 5H), the siRNA-treated groups failed to form a complete autophagosome although the phagophore had already been formed (Figure 5K, 5L, 5O, 5P, 5S and 5T).

## DISCUSSION

In this study, we explored the novel mechanisms of DBT in CT26 colorectal adenocarcinoma cells. DBT-PD induced autophagic death in CT26 cells, which was quite different from the traditional activity of DBT observed on the proliferation of hematopoietic [11–13] and osteoblast cells [14, 15] in the bone marrow. The mTOR/p70^S6K^ signaling pathway was an active part of the process.

Autophagy is a method of survival for cells, especially following radiation treatment for cancer. It may prevent tumor cells from death via apoptosis, which triggers the crosstalk between the autophagic and apoptotic signaling pathways [28, 29]. However, it is uncertain whether that is the complete role of autophagy. Many chemotherapy agents currently used result in the formation autophagosomes in tumor cells [30, 31]. For example, the treatment of MCF-7 breast cancer cells with tamoxifen resulted in an increase in the levels of autophagy and cell death, which could be inhibited by the addition of the class III-PI3K inhibitor 3-methyladenine that functions by blocking early stage autophagosome formation [30]. This indicated that a block in the formation of autophagic bodies was sufficient to decrease the antineoplastic effect of anti-estrogen drugs. Everolimus, an inhibitor of mTOR, blocked the effects caused by the loss of the TSC1/TSC2, induced autophagy, and reduced cell growth, proliferation, and angiogenesis. Everolimus sensitized Ras-transformed rat kidney epithelial RK3E cells to radiation *in vitro*, which mediated the radiosensitization associated with the autophagy pathway [32]. The inhibition of apoptosis (by a combination of Z-DEVD, a caspase-3 inhibitor, and everolimus) during radiotherapy resulted in improved radiation cytotoxicity compared with either agent alone in a xenograft model of H460 non-small cell lung cancer cells [33]. This allowed the exploration of the different roles of autophagy in cancer treatment.

A novel therapeutic target of mTOR inhibitors was used to control advanced cancers and prolong the survival time of cancer patients. Everolimus has been widely used in the treatment of breast and renal carcinoma. In a meta-analysis of randomized controlled trials, Qiao et al. summarized that the treatment of everolimus plus endocrine therapy in hormone receptor-positive and Her2/Neu receptor-negative patients caused adverse effects in patients with an approximate occurrence of 50% stomatitis, 40% rash, 33% nausea, 14% pneumonitis, 34% diarrhea, and 40% fatigue [34]; greater effects were also observed in addition to the increased injury. Temsirolimus is a specific inhibitor of mTOR, which interferes with the synthesis of proteins that regulate the proliferation, growth, and survival of tumor cells. It has also been used for the therapy of various cancers, in particular for renal cell carcinoma (RCC). A clinical trial of temsirolimus for RCC patients found the most common temsirolimus-related grade 3–4 adverse events were anemia (13%), hyperglycemia (9%), and asthenia (8%). Although pneumonitis occurred infrequently, the possible development of the condition should be monitored [35]. Systematic reviews and meta-analyses by Choueiri et al. demonstrated that the use of mTOR inhibitors was associated with a small but higher risk of fatal adverse events compared with control patients [36].

In both *in vitro* and *in vivo* studies, the presence of autophagosomes was consistently observed by using TEM after the treatment of CT26 cells with DBT. No obvious adverse effects on the liver, renal, or hematopoietic function were observed during studies in animal models. To obtain more firm support of our animal findings, future experiments with more abundant test mice/group are needed to support these initial findings. The p70^S6K^ protein is the substrate of mTORC1 complex, which is a key kinase involved in the regulation of cell growth [37]. Atg7 acts as an E1-like activating enzyme facilitating both microtubule-associated protein light chain 3 (LC3)-phosphatidylethanolamine and Atg12 conjugation during the elongation and enclosure processes of autophagosome formation. Thus, Atg7 stands at the hub of these two ubiquitin-like systems that involves LC3 and Atg12 in autophagic vesicle expansion [38]. Furthermore, the Atg7 protein was highly activated after the mTOR/p70^S6K^ signaling pathway was triggered in DBT-treated cancer cells. Similar to other mTOR inhibitor agents, DBT may offer a potential treatment option for patients with CRC who are resistant to standard treatments. However, there are some difficulties that still need to be overcome. Quality assurance (QA) and quality control (QC) are important in herbal medicines because the components inside the herbs always vary with the current environment and climate. The index components astragaloside IV and ferulic acid were detected and quantitated in our previous study. However, purified astragaloside IV (3.126 μg/mL) and ferulic acid (0.014 μg/mL) in amounts equivalent to that contained in 10 mg/mL DBT-PD had no significant growth inhibitory effect [21]. DBT is a Chinese herbal formula that has been traditionally used to treat menstrual anemia. Several literatures studied the effects of DBT, including antioxidant, immunomodulatory, anti-angiogenic activities. In all these studies, the activities of DBT were determined using DBT decoction. To date, there have been no reports on studies investigating the components of DBT effective for cancer therapy. Chemoradiotherapy is one of the most common modality for the treatment of colorectal cancer. We previously demonstrated that DBT promoted antitumor effect and sensitized colorectal cancer cells to chemoradiotherapy. Concurrent treatment of CT26 cells with DBT-PD and 5-fluorouracil (5-FU) markedly inhibited cell growth with a combination index (CI) of 0.42 after 72 h. Moreover, DBT-PD sensitized colorectal cancer cells to radiotherapy and concurrent chemoradiotherapy. This indicated that DBT could serve as a chemoradiosensitizer in colorectal cancer treatment [21]. The essential components of DBT that inhibit tumor growth should be purified and investigated further.

In this study, we explored the novel antineoplastic effect of the traditional Chinese formula DBT. An increase in the safety and efficiency of the approaches used to treat cancer patients is required; the novel application of traditional Chinese herbs might be a potential method to achieve this.

## MATERIALS AND METHODS

### Cell culture

The CT26 colorectal adenocarcinoma cell line was purchased from American Type Culture Collection (ATCC, Manassas, VA, USA). Cells were cultured in RPMI 1640 medium (GIBCO, Grand Island, NY, USA) supplemented with 10% heat-inactivated fetal calf serum (Hyclone, Logan, UT, USA), 100 IU/mL penicillin, 100 g/mL streptomycin, and 2 mM L-glutamine at 37°C in a humidified 5% CO_2_ incubator. The cells were passaged every 2–3 days and maintained in the exponential growth phase.

### Xenograft tumor implantation

Male BALB/c mice (6–8 weeks old) were obtained from the National Laboratory Animal Center (Taipei, Taiwan) and housed in a rodent facility under a 12-h light-dark cycle. Three groups, each containing three mice, were implanted with CT26 cells (2.5×10^5^ cells) via subcutaneous injection into the right gluteal region. Treatment was started after 10 days, when the tumors were approximately 0.5 cm in diameter. All experiments were performed in accordance with the regulations of the NIH Guide for the Care and Use of Laboratory Animals.

### Drug preparation

The contents of DBT, *Astragali* Radix (AR) and *Angelicae sinensis* Radix (ASR) in the ratio of 5:1, were kindly provided and authenticated by Chinese medicine manufacturer Sun Ten Pharmaceutical Co., Ltd, in Taiwan. Before extraction, *Angelicae sinensis* Radix was stir fried in rice wine (alcohol content, 75% v/v) and the pedicles were removed as per the traditional preparation method. AR and ASR were boiled in water for 1 h, and then separated into polysaccharide-enriched (DBT-PE) and polysaccharide-depleted (DBT-PD) fractions via precipitation in 50% ethanol. Both fractions were concentrated by freeze-drying. The purity of the indicator components of AR and ASR respectively, ferulic acid and astragaloside IV, were determined separately by HPLC-UV and HPLC-MS analysis and compared with the standard compounds of ferulic acid (ChromaDex, Irvine, CA, USA) and astragaloside IV (Tauto biotech, Shanghai, China) [21].

### Drug administration

After treatment with vehicle or 10 mg/mL DBT-PD for 48 h, CT26 cells were harvested for further investigation. For the *in vivo* assay, mice were orally administered the following treatments: 50% v/v alcohol (control group); 2.6 g/kg/day DBT dissolved in distilled water (DBT-treated group); and 0.39 g/kg/day DBT-PD dissolved in 50% v/v alcohol (DBT-PD-treated group) in 100 μL solution from day 1 to day 5 every week for 4 weeks consecutively [39].

### Evaluation of tumor volume, body weight, blood cell profiles, and biochemical parameters

The total body weight of each mouse and tumor size were measured every week by a single observer. The tumor volume was calculated using the formula: 0.5ab^2^, where a and b are the largest and smallest diameters, respectively. The number of leukocytes, erythrocytes, and thrombocytes count were estimated via retro-orbital blood sampling once a week after treatment and detected by Drew Hemavet HV950 (Drew Scientific, Inc., Miami Lake, FL, USA). Animals were killed 4 weeks after treatment. The plasma levels of alanine aminotransferase (ALT) and creatinine were also measured every week during the experimental period using a SYNCHRONLX20 spectrophotometer (Beckman Coulter, San Diego, CA, USA).

### Immunohistochemistry

Tumor specimens were harvested, fixed in 4% formalin for 48 h, embedded in paraffin, sectioned, deparaffinized in xylene, and rehydrated in ethanol. The samples were stained with hematoxylin and eosin for pathological examination. After antigen retrieval by boiling in 10 mM citrate buffer for 1 h, the sections were treated with 0.3% hydrogen peroxide in methanol to inactivate the endogenous peroxidase. Samples were incubated with a primary antibody to LC3B (1:100) at 4°C overnight, and then visualized using diaminobenzidine as the chromogen (VECTASTAIN ABC kit, Vector Laboratories, Burlingame, CA, USA). Hematoxylin was used as the counterstain. The slides were observed under an optical microscope (Olympus, Tokyo, Japan) at a magnification of 1000× and photographed with a charged-coupled device (CCD) camera. The slides were scanned using TissueFAXS Histo (Tissue Gnostic, Taborstraße, Vienna, Australia) with TissueFAXS imaging software (version4.2.6245.1019) and then analyzed by HistoQuest analysis software (version 4.0.4.0158) for counting the percentages of LC3B positive cells.

### Gene knockdown using small interfering RNA (siRNA) expression system

siRNAs specifically targeting Atg5 (MSS247019) and Atg7 (MSS292731) were purchased from ThermoFisher Scientific (Waltham, MA, USA). The siRNA sequences against Atg5 and Atg7 were GCGAGCAUCUGAGCUACCCAGAUAA and CAGCCUGGCAUUUGAUAAAUGUACA, respectively. Non-specific siRNA was used for a negative control in all gene-silencing experiments. The cells were transfected with control siRNA or siRNAs to Atg5, Atg7, or both using Lipofectamine RNAiMAX (Invitrogen, Waltham, MA, USA) in accordance with the manufacturer’s instructions for 24 h at 37°C in 5% CO_2_. The cells were subjected to drug treatment in fresh medium.

### Western blot

Treated cells were washed twice with ice-cold phosphate-buffered saline (PBS) and collected in lysis buffer (Cell Signaling Technology, Danvers, MA, USA). The cellular lysates were prepared and protein concentrations were determined using a bicinchoninic acid (BCA) assay kit (Pierce, Rockford, IL, USA). Equal amounts of proteins (50 μg) were separated via electrophoresis on a 10% SDS-polyacrylamide gel and then transferred onto a nylon membrane for western blot analysis. Specific antibodies for microtubule-associated light chain protein 3B (LC3B)-I, LC3B-II, p70^S6K^, phospho-p70^S6K^, Beclin-1, Atg5, Atg7, and p62 were used to detect the levels of LC3B-I, LC3B-II, p70^S6K^, phospho-p70^S6K^, Beclin-1, Atg5, Atg7, and p62 respectively. Immunoreactive proteins were visualized using enhanced chemiluminescence detection reagent (Amersham Pharmacia, Piscataway, NJ, USA), and β-actin was used as the internal control. Multi Gauge V2.1 software was used to assess the ratio of LC3B-II/LC3B-I proteins, which revealed the transforming rate from LC3B-I to LC3B-II proteins.

### Confocal microscopy

Cells were seeded onto coverslips and treated as described above. After treatment, the cells were washed with PBS, fixed in 4% paraformaldehyde, and permeabilized with 0.5% Triton X-100. The slides were incubated at 4°C overnight with the primary antibody anti-LC3B (1:200, Cell Signaling Technology) and then with rhodamine-conjugated secondary antibody at room temperature for 1 h. The nuclei of cells were stained with 4', 6-diamidino-2-phenylindole and cells were observed by Leica TCS SP5 confocal fluorescence microscope (Wetzlar, Germany). Images were captured with the Leica DC 300F charged-coupled device camera at a magnification of 400×.

### Transmission electron microscope (TEM)

After treatment, the cells were collected and washed. For animal, mice were sacrificed and tumor specimens were removed and fixed in 4% formaldehyde. The specimens were then diced into 1 mm^3^ pieces. Cells and tissues were fixed with 2.5% glutaraldehyde in 0.1 M cacodylate buffer for 2 h. Samples were then washed, post-fixed with 1% osmium tetroxide for 1 h, and then dehydrated with alcohol and acetone. The samples were embedded into Spurr’s resin, semithin-sectioned, stained with 0.5% toluidine blue, and examined under a light microscope (BX51, Olympus, Tokyo, Japan). Ultrathin sections were stained with 5% uranylacetate and Reynold’s lead citrate. The morphology of samples was observed and photographed using a transmission electron microscope (JEM-1200EXII, JEOL Co., Tokyo, Japan).

### Statistical analysis

Data were expressed as the mean ± SE or percentage. Results were analyzed with repeated measured ANOVA with Fisher’s LSD method to compare tumor size, body weight, ALT and creatinine levels, and leukocyte, erythrocyte, thrombocyte count, and percentage of LC3B positive cells between the treatment and control groups. *p* values <0.05 were considered statistically significant.

## Abbreviations

ALT: alanine aminotransferase
AR: *Astragali* Radix
ASR: *Angelicae sinensis* Radix
BCA: bicinchoninic acid
CRC: colorectal cancer
DBT: Danggui Buxue Tang
DBT-PD: Danggui Buxue Tang - polysaccharide depleted
PBS: phosphate buffered saline
RCC: renal cell carcinoma
siRNA: small interfering RNA
TEM: transmission electron microscopy

## References

[1] Torre LA, Bray F, Siegel RL, Ferlay J, Lortet-Tieulent J, Jemal A (2015). Global cancer statistics, 2012. CA Cancer J Clin.

[2] Eddy DM (1990). Screening for colorectal cancer. Ann Intern Med.

[3] Siegel RL, Miller KD, Jemal A (2016). Cancer statistics, 2016. CA Cancer J Clin.

[4] Center MM, Jemal A, Smith RA, Ward E (2009). Worldwide variations in colorectal cancer. CA Cancer J Clin.

[5] O'Connell JB, Maggard MA, Ko CY (2004). Colon cancer survival rates with the new American Joint Committee on Cancer sixth edition staging. J Natl Cancer Inst.

[6] Hammond WA, Swaika A, Mody K (2016). Pharmacologic resistance in colorectal cancer: a review. Ther Adv Med Oncol.

[7] Lee SY, Oh SC (2016). Advances of targeted therapy in treatment of unresectable metastatic colorectal cancer. Biomed Res Int.

[8] Easton JB, Houghton PJ (2006). mTOR and cancer therapy. Oncogene.

[9] Huang Z, Wu Y, Zhou X, Qian J, Zhu W, Shu Y, Liu P (2015). Clinical efficacy of mTOR inhibitors in solid tumors: a systematic review. Future Oncol.

[10] Zhang WL, Zheng KY, Zhu KY, Zhan JY, Bi CW, Chen JP, Dong TT, Choi RC, Lau DT, Tsim KW (2013). Chemical and biological assessment of angelica roots from different cultivated regions in a chinese herbal decoction danggui buxue tang. Evid Based Complement Alternat Med.

[11] Gao QT, Cheung JK, Choi RC, Cheung AW, Li J, Jiang ZY, Duan R, Zhao KJ, Ding AW, Dong TT, Tsim KW (2008). A Chinese herbal decoction prepared from Radix Astragali and Radix Angelicae Sinensis induces the expression of erythropoietin in cultured Hep3B cells. Planta Med.

[12] Gao QT, Cheung JK, Li J, Chu GK, Duan R, Cheung AW, Zhao KJ, Dong TT, Tsim KW (2006). A Chinese herbal decoction, Danggui Buxue Tang, prepared from Radix Astragali and Radix Angelicae Sinensis stimulates the immune responses. Planta Med.

[13] Yang M, Chan GC, Deng R, Ng MH, Cheng SW, Lau CP, Ye JY, Wang L, Liu C (2009). An herbal decoction of Radix astragali and Radix angelicae sinensis promotes hematopoiesis and thrombopoiesis. J Ethnopharmacol.

[14] Choi RC, Gao QT, Cheung AW, Zhu JT, Lau FT, Li J, Li WZ, Chu GK, Duan R, Cheung JK, Ding AW, Zhao KJ, Dong TT, Tsim KW (2011). A chinese herbal decoction, danggui buxue tang, stimulates proliferation, differentiation and gene expression of cultured osteosarcoma cells: genomic approach to reveal specific gene activation. Evid Based Complement Alternat Med.

[15] Wang WL, Sheu SY, Chen YS, Kao ST, Fu YT, Kuo TF, Chen KY, Yao CH (2014). Evaluating the bone tissue regeneration capability of the Chinese herbal decoction Danggui Buxue Tang from a molecular biology perspective. Biomed Res Int.

[16] Gao QT, Choi RC, Cheung AW, Zhu JT, Li J, Chu GK, Duan R, Cheung JK, Jiang ZY, Dong XB, Zhao KJ, Dong TT, Tsim KW (2007). Danggui buxue tang--a Chinese herbal decoction activates the phosphorylations of extracellular signal-regulated kinase and estrogen receptor alpha in cultured MCF-7 cells. FEBS Lett.

[17] Wang CC, Cheng KF, Lo WM, Law C, Li L, Leung PC, Chung TK, Haines CJ (2013). A randomized, double-blind, multiple-dose escalation study of a Chinese herbal medicine preparation (Dang Gui Buxue Tang) for moderate to severe menopausal symptoms and quality of life in postmenopausal women. Menopause.

[18] Gao J, Huang Y, Li P, Xu D, Li J, Liu Y, Huang Z, Wu Q, Shao X (2011). Antifibrosis effects of total glucosides of Danggui-Buxue-Tang in a rat model of bleomycin-induced pulmonary fibrosis. J Ethnopharmacol.

[19] Lv J, Zhao Z, Chen Y, Wang Q, Tao Y, Yang L, Fan TP, Liu C (2012). The chinese herbal decoction danggui buxue tang inhibits angiogenesis in a rat model of liver fibrosis. Evid Based Complement Alternat Med.

[20] Chen Y, Chen Q, Lu J, Li FH, Tao YY, Liu CH (2009). Effects of Danggui Buxue Decoction () on lipid peroxidation and MMP-2/9 activities of fibrotic liver in rats. Chin J Integr Med.

[21] Chen ST, Lee TY, Tsai TH, Lin YC, Lin CP, Shieh HR, Hsu ML, Chi CW, Lee MC, Chang HH, Chen YJ (2016). The traditional Chinese medicine DangguiBuxue Tang sensitizes colorectal cancer cells to chemoradiotherapy. Molecules.

[22] Klionsky DJ, Abeliovich H, Agostinis P, Agrawal DK, Aliev G, Askew DS, Baba M, Baehrecke EH, Bahr BA, Ballabio A, Bamber BA, Bassham DC, Bergamini E (2008). Guidelines for the use and interpretation of assays for monitoring autophagy in higher eukaryotes. Autophagy.

[23] Efeyan A, Comb WC, Sabatini DM (2015). Nutrient-sensing mechanisms and pathways. Nature.

[24] Bellot G, Garcia-Medina R, Gounon P, Chiche J, Roux D, Pouyssegur J, Mazure NM (2009). Hypoxia-induced autophagy is mediated through hypoxia-inducible factor induction of BNIP3 and BNIP3L via their BH3 domains. Mol Cell Biol.

[25] Pyo JO, Nah J, Jung YK (2012). Molecules and their functions in autophagy. Exp Mol Med.

[26] Sou YS, Waguri S, Iwata J, Ueno T, Fujimura T, Hara T, Sawada N, Yamada A, Mizushima N, Uchiyama Y, Kominami E, Tanaka K, Komatsu M (2008). The Atg8 conjugation system is indispensable for proper development of autophagic isolation membranes in mice. Mol Biol Cell.

[27] Moscat J, Diaz-Meco MT (2009). p62 at the crossroads of autophagy, apoptosis, and cancer. Cell.

[28] Rikiishi H (2012). Novel insights into the interplay between apoptosis and autophagy. Int J Cell Biol.

[29] Su M, Mei Y, Sinha S (2013). Role of the crosstalk between autophagy and apoptosis in cancer. J Oncol.

[30] Bursch W, Ellinger A, Kienzl H, Torok L, Pandey S, Sikorska M, Walker R, Hermann RS (1996). Active cell death induced by the anti-estrogens tamoxifen and ICI 164 384 in human mammary carcinoma cells (MCF-7) in culture: the role of autophagy. Carcinogenesis.

[31] Kanzawa T, Germano IM, Komata T, Ito H, Kondo Y, Kondo S (2004). Role of autophagy in temozolomide-induced cytotoxicity for malignant glioma cells. Cell Death Differ.

[32] Su YC, Yu CC, Hsu FT, Fu SL, Hwang JJ, Hung LC, Lee MS, Chiou WY, Lin HY, Hung SK (2014). Everolimus sensitizes Ras-transformed cells to radiation *in vitro* through the autophagy pathway. Int J Mol Med.

[33] Kim KW, Hwang M, Moretti L, Jaboin JJ, Cha YI, Lu B (2008). Autophagy upregulation by inhibitors of caspase-3 and mTOR enhances radiotherapy in a mouse model of lung cancer. Autophagy.

[34] Qiao L, Liang Y, Mira RR, Lu Y, Gu J, Zheng Q (2014). Mammalian target of rapamycin (mTOR) inhibitors and combined chemotherapy in breast cancer: a meta-analysis of randomized controlled trials. Int J Clin Exp Med.

[35] Bellmunt J, Szczylik C, Feingold J, Strahs A, Berkenblit A (2008). Temsirolimus safety profile and management of toxic effects in patients with advanced renal cell carcinoma and poor prognostic features. Ann Oncol.

[36] Choueiri TK, Je Y, Sonpavde G, Richards CJ, Galsky MD, Nguyen PL, Schutz F, Heng DY, Kaymakcalan MD (2013). Incidence and risk of treatment-related mortality in cancer patients treated with the mammalian target of rapamycin inhibitors. Ann Oncol.

[37] Steelman LS, Martelli AM, Cocco L, Libra M, Nicoletti F, Abrams SL, McCubrey JA (2016). The therapeutic potential of mTOR inhibitors in breast cancer. Br J Clin Pharmacol.

[38] Xiong J (2015). Atg7 in development and disease: panacea or Pandora's Box?. Protein Cell.

[39] Nair AB, Jacob S (2016). A simple practice guide for dose conversion between animals and human. J Basic Clin Pharm.

